# The right to data protection and the COVID-19 pandemic: the European approach

**DOI:** 10.1007/s12027-020-00644-4

**Published:** 2020-12-07

**Authors:** Magdalena Kędzior

**Affiliations:** ERA, Trier, Germany

## Introduction and background

2020 has been marked by the unprecedented outbreak of the COVID-19 pandemic. Its complex impact on EU policy-making and numerous implications for the EU legal order have been reflected in the content of nearly all events organised by ERA in the second half of 2020. The pandemic created a need for organisations – including the EU institutions – to reinvent their activities by adopting additional safety measures, by allowing employees to work from home and/or by switching to an entirely online working environment. Preventive measures adopted by organisations happen to include the processing of information concerning the movement of employees, such as location data, as well as concerning one of the most vulnerable categories of data, which is health data.

Against the background of these developments since the outbreak of the coronavirus pandemic, Member States, backed by the European Commission, have been assessing the effectiveness, security, privacy, and data protection aspects of digital solutions to address the crisis. The question instinctively posed has been whether the GDPR and other data protection laws^1^ hinder the processing of personal data, considered necessary to fight the pandemic, and if so, then under which conditions.^2^

The reflections that follow touch upon major legal challenges that have been identified on the EU level with regard to safeguarding privacy when fighting the pandemic and the approach taken to these challenges by the EU institutions. This contribution aims therefore to map the EU’s approach to COVID-19 with regard to privacy and data protection, to identify the areas that cooperation at European level has been focusing upon and to determine the added value thereof. Beyond this, the question of what might have been the possible obstacles to a more harmonised approach towards fighting the pandemic in the EU Member States will also be considered.

## Legal challenges regarding data protection rights

### General remarks

Measures undertaken to fight and protect against COVID-19 have given rise to significant legal implications for the rights and freedoms of individuals. Discussions and controversies focused around several topics such as the processing of employees’ personal data (including health data) by employers; workplace monitoring; the processing of health data by public authorities; the data protection requirements needed to be met by trustworthy and efficient apps; the tracing of location data; as well as data subjects’ rights in connection with states of emergency in Member States.

As for the collection and processing of employees’ personal data (including health data) by employers, one should bear in mind that some issues relating to data protection in the context of employment – like the monitoring of employees – may be subject to regulation at national level. Art. 88 GDPR allows Member States to provide for more specific rules to ensure the protection of rights and freedoms in respect of the processing of employees’ personal data.^3^ Consequently, the approach taken by organisations might differ from one Member State to another. Relevant guidance for the EU institutions as employers has been issued by the European Data Protection Supervisor.^4^

With regard to teleworking, the Council of Europe has underlined that this should not lead to the monitoring of employees, including by means of video. Instead non-intrusive measures should be applied.^5^ With reference to sensitive data processing, including health related data, employers should respect the principles of necessity, proportionality and accountability and should also be guided by principles designed to minimise any risks that such processing might pose to employees’ rights and fundamental freedoms, in particular their right to privacy as elaborated in Recommendation CM/Rec(2015)5 on the processing of personal data in the context of employment.

As regards the processing of health-related data by public authorities, relevant recommendations have been issued by the Council of Europe.^6^ It has been stressed that communications to the public by health and government authorities should remain a priority in order to protect, inform and advise the public. Nonetheless, during such communications, the publication of sensitive data (such as health-related data) of specific individuals should be avoided and it is recommended that the processing of such data is only done if additional technical and organisational measures complementing those applied to non-sensitive data are put in place.

### Issues addressed within the EU context

In the EU context, particular attention has been paid to the development and deployment of contact tracing apps and the tracking of location data. Tracing and warning apps can help break the chain of coronavirus infections and save lives by complementing manual tracing. Most Member States have launched national contact-tracing and warning applications. In addition, the Member States, together with the Commission, have set up a service allowing national apps to communicate with each other across borders in Europe, so that users can install a unique App which will allow them to be warned if they were in contact with someone who has indicated that they have tested positive for COVID-19.^7^ The Commission has ensured that contact tracing and warning apps are only used voluntarily, are based on Bluetooth proximity technology, respect users’ privacy and do not enable the tracking of people’s locations.^8^

On 8 April 2020, the European Commission issued Recommendation 2020/518^9^, proposing a number of steps and measures for developing a common approach to the use of mobile applications and mobile data in response to the coronavirus pandemic across the European Union. The purpose of the Recommendation was to support the gradual lifting of coronavirus containment measures by using mobile data and apps, and provided key principles for the use of mobile applications used for social distancing measures, for warning, for prevention and for contact tracing. The Commission stressed that any use of apps and data should respect data security and EU fundamental rights, such as privacy and data protection.

The Recommendation was accompanied by Guidance on Apps supporting the fight against COVID-19 pandemic in relation to data protection (2020/C 124 I/01).^10^ The framework developed by the Commission provides that tracing apps must be voluntary, transparent, temporary, cybersecure, and use temporary and pseudonymised data. They should rely on Bluetooth technology and be approved by national health authorities, and be interoperable across borders as well as across operating systems. The European Data Protection Board took a similar approach in its Guideline 04/2020 of 21 April 2020.^11^ The EDPB noted that in deploying contract tracing apps general data protection principles – such as lawfulness, purpose limitation, data minimisation, accuracy, storage limitation, integrity and confidentiality as well as accountability – should be applied.^12^

The European Data Protection Supervisor issued a statement in which he called for a pan-European “COVID-19 mobile application” model, coordinated at EU level. It stressed that ideally, coordination with the World Health Organisation should also take place, so as to ensure data protection globally by design from the start. The EDPS underlined that, the use of temporary broadcast identifiers and Bluetooth technology for contact tracing seemed to be a useful path towards achieving privacy and personal data protection effectively.^13^ With reference to the use of location data for mapping the spread of the pandemic, the European Data Protection Supervisor underlined that effectively-anonymised data fall outside the scope of data protection rules and underlined the importance of applying adequate measures to ensure the secure transmission of data from telecom providers. Regarding data retention, it welcomed the idea that the data obtained from mobile operators would be deleted as soon as the current emergency came to an end. It had also been made clear that these special services had been deployed because of the specific crisis and would be of a temporary character. The European Data Protection Supervisor noted that such developments usually do not allow for the possibility of stepping back when the emergency is gone and that such a solution should be still recognised as extraordinary.^14^ The European Data Protection Board in its Guideline 04/2020 on the use of location data and contact tracing tools in the context of the Covid-19 outbreak of 21 April emphasised that the GDPR and Directive 2002/58/EC both contain specific rules allowing for the use of anonymous or personal data to support public authorities and other actors at national and EU levels in monitoring and containing the spread of the virus. The European Data Protection Board recalled that location data collected from electronic communication providers may only be transmitted to authorities or other third parties if it has been anonymised. Where data indicates the geographic position of the terminal equipment of a user, the consent of the user is required in such a case. As for information, including location data, collected directly from the users’ terminal equipment, the storing of information on the user’s device or the gaining of access to the information already stored is allowed only if the user has given consent or storage and/or access is necessary for the service requested by the user.

Regarding the processing of personal data concerning health for the purposes of scientific research in the context of the COVID-19 outbreak, the European Data Protection Board adopted Guideline 03/2020.^15^ In this context, the European Data Protection Board recalled that the GDPR provides special rules for the processing of health data for the purposes of scientific research that are also applicable in the context of the COVID-19 pandemic. According to Article (9) (2) (i) and (j) GDPR, the national legislator of each Member State may enact laws to enable the processing of health data for scientific research purposes. Therefore, the conditions and the extent for such processing may vary depending on the laws enacted by the particular Member State. Interestingly, the European Data Protection Board noted that, in principle, situations as the COVID-19 outbreak do not suspend or restrict the possibility of data subjects exercising their rights. However, Article 89 (2) GDPR allows national legislators to restrict (some) of the data subject’s rights within processing that is carried out for scientific purposes. Because of this, the restrictions of the rights of data subjects may also vary depending on the laws enacted in the particular Member State.

## Institutional responses to data protection in times of COVID-19

The action undertaken by the EU institutions, is to be seen against the background of the whole range of responses to data protection in the time of the COVID-19 pandemic. Already on 17 March 2020, the Executive Committee of the Global Privacy Assembly (GPA) recognised the unprecedented challenges being faced in addressing the spread of Coronavirus (COVID-19) and stressed that addressing these challenges required coordinated responses at national and global levels, including the sharing of personal information as necessary by organisations and governments, as well as across borders. As highlighted in its statement,

“health data is considered sensitive across many jurisdictions, but work between data protection authorities and governments means we have already seen many examples of national approaches to sharing public health messages; of using the latest technology to facilitate safe and speedy consultations and diagnoses; and of creating linkages between public data systems to facilitate identification of the spread of the virus.”^16^

The OECD, in its policy response of 14 April 2020 to Coronavirus (COVID-19) entitled *Ensuring data privacy as we battle COVID-19* stated that some digital responses to the crisis had precipitated novel data governance and privacy challenges. It underlined that only a few countries had frameworks in place to support the extraordinary contact-tracing and population-wide surveillance measures. It correctly noted that privacy enforcement authorities had a key role to play as governments enacted emergency legislation and data controllers sought legal certainty.^17^

The European Union Fundamental Rights Agency (FRA) undertook extensive studies scrutinising the fundamental rights implications of the COVID-19 pandemic. With reference to privacy and data protection, a bulletin dedicated to contact-tracing apps has been published.^18^ The evidence collected by the Agency shows that nearly all EU data protection authorities issued guidelines relating to the pandemic. These statements reaffirm that the rights to health and the protection of personal data go hand in hand. They also underline that any measure that would infringe the rights to a private life and to data protection should be grounded in law, be necessary and be proportionate.^19^

A number of civil society organisations such as EDRI^20^ and AccessNow^21^ issued comprehensive statements and policy recommendations on data protection requirements in fighting the pandemic. Interestingly, the European Law Institute (ELI) also drafted principles for the COVID-19 crisis which address some of the most important legal issues arising in relation to the pandemic, offering guidance to the Data Protection Commissioner of the Council of Europe on how to steer through these unprecedented times.^22^

The Council of Europe in a joint statement of 30 March 2020 on the right to data protection in the context of the COVID-19 pandemic recalled that data protection can in no manner be an obstacle to saving lives and that the applicable principles always allow for a balancing of the interests at stake.^23^ As regards the lawfulness of personal data processing, it stressed that the processing of data can be carried out either on the basis of the data subject’s consent or some other legitimate basis laid down by law. As explicitly provided by the Explanatory Report to Convention 108+, such a legitimate basis notably encompasses data processing that is necessary to protect the vital interests of individuals, and data processing that is carried out on the grounds of public interest, such as for the purposes of monitoring a life-threatening epidemic. According to Article 11 of Convention 108 +, exceptions to the right to data protection are to be

“provided for by law, respect the essence of the fundamental rights and freedoms and constitute a necessary and proportionate measure in a democratic society. Where restrictions are being applied, those measures have to be taken solely on a provisional basis and only for a period of time explicitly limited to the state of emergency”.

Data protection authorities have been invited to carefully assess the measures taken by state authorities by reference to those conditions.^24^

At EU level, the European Data Protection Board adopted a formal statement on the processing of personal data in the context of the COVID-19 outbreak on 19 March 2020.^25^ The Board noted that data protection rules (such as the GDPR) do not hinder measures taken in the fight against the coronavirus pandemic and that it is in the interests of humanity to curb the spread of diseases and to use modern techniques in the fight against them. The Board stated that personal data must nonetheless be processed in a lawful manner, that the principle of minimisation must be observed and that processed personal data must be properly secured. The European Data Protection Board confirmed that, in line with Recital 46 of the GDPR (which expressly refers to the monitoring of epidemics), the collection and further processing of health-related data might serve both the public interest in the area of public health and protect the vital interests of data subjects. Still, the specific legal basis for such processing is mostly a matter of national law, especially in the context of employment.

Guidance on issues relating to data protection and the use of tracking and geolocation tools in the context of the COVID-19 outbreak has been identified as a priority at European Data Protection Board level.^26^ In subsequent steps, guidance on issues relating to data protection and the processing of health data in the context of the COVID-19 outbreak for scientific research purposes;^27^a statement on restrictions on data subject rights in connection to the state of emergency in Member States;^28^ a statement on the data protection impact of the interoperability of contact tracing apps;^29^ and a statement on the processing of personal data in the context of the reopening of borders following the COVID-19 outbreak^30^ were all adopted. Relevant guidelines were also made available by national data protection regulators, some of which limited themselves, however, to publishing the European Data Protection Board statements.

In defining its approach to data protection in times of the coronavirus pandemic, the European Data Protection Supervisor has worked closely with the European Data Protection Board. On 6 April 2020, the European Data Protection Supervisor issued a call for a pan-European approach against the pandemic.^31^ Acting as supervisory body for data protection in EU institutions, the European Data Protection Supervisor adopted a document *“Orientations from the EDPS. Reactions of EU institutions as employers to the COVID-19 crisis”* on 15 July 2020. The paper compiles advice given on questions such as teleworking tools, staff management, health data issues and replying to data subject access requests.^32^ The European Data Protection Supervisor noted that data protection rules currently in force within the EU institutions are flexible enough to allow for various measures in order to render possible the continuity of EU institutions’ operations. Notwithstanding this however, there should be no doubt that the essential data protection requirements set out in Article 8 of the EU Charter of Fundamental Rights and in Regulation (EU) 2018/1725 (such as the principles of accountability, data protection by design and by default, security and transparency) continued to apply. Within its mandate, the European Data Protection Supervisor has been consulted by the European Institutions (in particular by the European Commission) on the monitoring of the spread of COVID-19 and has issued relevant guidance letters in this regard.^33^ The necessity for the urgent establishment of a coordinated European approach to handle the emergency in the most efficient, effective and compliant way possible and the clear need to act at the European level have been emphasised.

As indicated above the European Commission‘s involvement has related mainly to recommendations on a common Union toolbox for the use of technology and data to combat and exit from the COVID-19 crisis, in particular concerning mobile applications and the use of anonymised mobility data. Member States in the eHealth Network, backed by the Commission, adopted an EU toolbox 34 on contact tracing applications in the EU fight against COVID-19, setting out the foundations of a common pan-European approach to contact tracing and warning apps. In addition, the eHealth Network adopted interoperability guidelines^35^ on 13 May 2020. Finally, in June 2020, the eHealth Network adopted technical specifications^36^ and guidelines^37^ establishing the architecture for a Federation Gateway Service which would allow the exchange of contact tracing keys between Member States. The modalities for processing personal data in the Federation Gateway were adopted in July with the amendment of the Implementing Decision on the eHealth Network.^38^ The development and deployment of the Federation Gateway was completed by the end of September 2020.^39^

Beyond this, the European Commission issued Guidelines on COVID-19 *in vitro* diagnostic tests and their performance.^40^ This document outlines the regulatory and market-relevant context of COVID-19-related *in vitro* diagnostic testing devices in the EU and gives an overview of different types of tests and their purposes. It includes considerations on device performance and validating that performance and sets out elements to be considered by Member States in defining national strategies, and by economic operators in placing devices on the market, all with the objective of ensuring that safe and effective devices for COVID-19-related testing are available in the EU.

## EU data protection laws: an obstacle in fighting the pandemic?

Any concerns regarding whether the GDPR and other data protection laws would hinder the processing of personal data for the purposes of undertaking measures aimed at fighting the pandemic, such as contact tracing, have been dispelled by the European Data Protection Board 41 and the European Data Protection Supervisor.^42^ The European Data Protection Board recalled that the GDPR foresees derogations to the prohibition of processing of certain special categories of personal data, such as health data, where this is necessary for reasons of substantial public interest in the area of public health (Art. 9.2.i), on the basis of Union or national law, or where there is a need to protect the vital interests of the data subject (Art. 9.2.c), while recital 46 explicitly refers to the control of an epidemic.

The European Data Protection Supervisor has noted that the right to the protection of personal data is not an absolute right; it must be considered in relation to its function in society and be balanced against other fundamental rights, in accordance with the principle of proportionality. As already mentioned above, legality of data processing, even sensitive data, like data about health, can be safeguarded when processing is necessary for reasons of substantial public interest, on the basis of Union or Member State law which should however be proportionate to the aim pursued. The European Data Protection Supervisor also confirmed that the GDPR allows for the processing of sensitive data when this is necessary for reasons of public interest in the area of public health, such as protecting against serious cross-border threats to health. As already indicated, the conditions required in order to process lawfully traffic and location data have been set by national laws implementing the Directive on Privacy and Electronic Communications. According to the ePrivacy Directive, exceptional legislative measures adopted by the Member States can restrict the scope of the rights and obligations provided by the ePrivacy regime. These national legislative measures should have the sole purpose of safeguarding public security and should only allow restrictions that constitute a necessary, appropriate and proportionate measure within a democratic society.

## Conclusions

Faced with challenges concerning the fundamental rights to privacy and to data protection, the EU institutions have taken note of some of the problems that have arisen in times of the COVID-19 pandemic and recalled the applicable standards. EU data protection experts have underlined how data protection rules, in particular the GDPR, should not hinder the measures that need to be implemented in the fight against the COVID-19 pandemic. As has been shown, the EU data protection legal framework was designed to be sufficiently flexible and as such, is able to allow for both an efficient response in limiting the pandemic and for protecting fundamental human rights and freedoms. By adopting numerous policy and guidelines documents, the EU institutions have proven themselves able to preserve privacy in emergency situations and even launch innovative technical solutions which fight the pandemic, while applying principles of personal data protection.

Without any doubt, an element of added value to the efforts undertaken at EU level has been the adoption on measures on the interoperability of contract tracing apps which came about through a Commission decision. As is so often the case, the EU legislator addressed in this case issues of common interest to EU citizens in cross-border situations, paying due attention to privacy and data protection matters. These efforts should by no means be underestimated. Wojciech Wiewiórowski (the European Data Protection Supervisor) rightly stated in this context that

“the crisis will not be finished in weeks. It will take months to fight it and years to recover. Given that we are so connected with each other, we will not be able to solve it with national tools only. The more European our answer is, the better the results we will achieve.”

Action taken on national level has however been not fully harmonised. There seem to be several reasons for that. Some aspects of personal data protection law, like data protection in the context of employment (e.g., health and safety), may be subject to more specific national regulation. In particular, the collection of health-related data is mostly a matter of national law, especially in the context of employment. The area of data protection in electronic communications is still governed by the E-Privacy Directive, subject to national implementation and the restrictions of the rights of data subjects may vary depending on the laws of the particular Member State. Finally, bearing in mind that in the area of public health, EU institutions have limited legislative competence, it is mostly non-binding acts such as recommendations that have been deployed. Whether or not such measures, implemented at national level, have been successful from a public health perspective, should be carefully monitored and studied in order to learn lessons for the future.

## Footnotes

1 Directive 2002/58 on the processing of personal data and the protection of privacy in the electronic communications sector. OJ L201, of 2002-07-31, pp. 37 – 47, https://eur-lex.europa.eu/legal-content/EN/TXT/PDF/?uri=CELEX:32002L0058&from=EN [10.10.2020].

2 *E. Ventrella*, *Privacy in emergency circumstances: Data Protection and the COVID-19 pandemic*, ERA Forum 3/2020, p. 2. One should bear in mind that any measure restricting data protection rights in the EU should be in line with the European Charter of Fundamental Rights (Art. 52 (1)), the General Data Protection Regulation and the European Convention on Human Rights (Art. 8 (2)).

3 Rec. 155 GDPR.

4 Orientations from the European Data Protection Supervisor: Reactions of EU institutions as employers to the COVID-19 crisis, 15 July 2020, https://edps.europa.eu/sites/edp/files/publication/20-07-15_edps_guidelines_remote_work_en.pdf [30 July 2020].

5 Coronavirus pandemic in the EU – Fundamental Rights Implications – Bulletin # 1, FRA, February 2020 – March 2020, p. 42, https://fra.europa.eu/sites/default/files/fra_uploads/fra-2020-coronavirus-pandemic-eu-bulletin_en.pdf [10 September 2020].

6 Protection of health-related data – Recommendation CM/Rec(2019)2 adopted by the Committee of Ministers of the Council of Europe 27 March 2019. https://edoc.coe.int/en/international-law/7969-protection-of-health-related-date-recommendation-cmrec20192.html [20 July 2020]. Joined statement on the right to data protection in the context of the COVID-19 pandemic, Strasbourg 30 March 2020: https://rm.coe.int/covid19-joint-statement/16809e09f4 [10 July 2020].

7 Currently, the service is “decentralised” and the calculations are carried out in the user’s app. It has been adopted by the majority of Member States. Three national apps (Germany, Ireland, and Italy) were first linked on 19 October 2020, when the system came online. https://ec.europa.eu/info/live-work-travel-eu/health/coronavirus-response/travel-during-coronavirus-pandemic/how-tracing-and-warning-apps-can-help-during-pandemic_en#:~:text=lives%20and%20livelihoods-,Tracing%20and%20warning%20apps%20can%20help%20break%20the%20chain%20of,does%20not%20stop%20at%20borders [7 November 2020].

8 *Op. cit*.

9 Commission Recommendation (EU) 2020/518 of 8 April 2020 on a common Union toolbox for the use of technology and data to combat and exit from the COVID- 19 crisis, in particular concerning mobile applications and the use of anonymised mobility data, OJ (EU) L 114/7, https://eur-lex.europa.eu/legal-content/EN/TXT/PDF/?uri=CELEX:32020H0518&from=ENendation%202020/510 [15 July 2020].

10 Communication from the Commission: Guidance on Apps supporting the fight against COVID-19 pandemic in relation to data protection 2020/C 124 I/01. https://eur-lex.europa.eu/legal-content/EN/TXT/PDF/?uri=CELEX:52020XC0417(08)&from=EN [15 July 2020].

11 Guidelines 04/2020 on the use of location data and contact tracing tools in the context of the COVID-19 outbreak Adopted on 21 April 2020, https://edpb.europa.eu/sites/edpb/files/files/file1/edpb_guidelines_20200420_contact_tracing_covid_with_annex_en.pdf [20 July 2020].

12 *E. Ventrella*, *op. cit*.

13 *W. Wiewiórowski*, EU Digital Solidarity: a call for a pan-European approach against the pandemic, 6 April 2020, https://edps.europa.eu/sites/edp/files/publication/2020-04-06_eu_digital_solidarity_covid19_en.pdf [10 July 2020].

14 European Data Protection Supervisor Comments on the European Commission’s plan to access telecommunications data from telecommunications service providers to monitor the COVID-19 spread (of 25 March 2020). https://edps.europa.eu/sites/edp/files/publication/20-03-25_edps_comments_concerning_covid-19_monitoring_of_spread_en.pdf [30 June 2020].

15 Guidelines 03/2020 on the processing of data concerning health for the purpose of scientific research in the context of the COVID-19 outbreak Adopted on 21 April 2020, https://edpb.europa.eu/sites/edpb/files/files/file1/edpb_guidelines_202003_healthdatascientificresearchcovid19_en.pdf [15 September 2020].

16 Statement by the GPA Executive Committee on the Coronavirus (COVID-19) pandemic of 17 March 2020, https://globalprivacyassembly.org/gpaexco-covid19 [30 July 2020].

17 OECD Policy Responses to Coronavirus (COVID-19), Ensuring data privacy as we battle COVID-19, Version of 14 April 2020, http://www.oecd.org/coronavirus/policy-responses/ensuring-data-privacy-as-we-battle-covid-19-36c2f31e/#section-d1e690 [20 July 2020].

18 Coronavirus pandemic in the EU – Fundamental Rights Implications: with a focus on contact tracing apps – Bulletin # 2, FRA, March 2020 – April 2020 https://fra.europa.eu/sites/default/files/fra_uploads/fra-2020-coronavirus-pandemic-eu-bulletin_en.pdf [20 July 2020].

19 *Op. cit*.

20 European Digital Rights EDRI calls for fundamental rights-based responses to COVID-19, https://edri.org/covid19-edri-coronavirus-fundamentalrights/ [14 July 2020].

21 Recommendation on privacy and data protection in the fight against COVID-19, https://www.accessnow.org/cms/assets/uploads/2020/03/Access-Now-recommendations-on-Covid-and-data-protection-and-privacy.pdf [16 July 2020].

22 https://www.europeanlawinstitute.eu/fileadmin/user_upload/p_eli/Publications/ELI_Principles_for_the_COVID-19_Crisis.pdf [15 July 2020].

23 As is noted in the statement, “in accordance with Convention 108+ it is crucial, that even in particularly difficult situations, data protection principles are respected and therefore it is ensured that data subjects are made aware of the processing of personal data related to them; processing of personal data is carried out only if necessary and proportionate to the explicit, specified and legitimate purpose pursued; an impact assessment is carried out before the processing is started; privacy by design is ensured and appropriate measures are adopted to protect the security of data, in particular when related to special categories of data such as health related data; data subjects are entitled to exercise their rights.” Joint Statement of 30 March 2020, op. cit., https://rm.coe.int/covid19-joint-statement/16809e09f4 [20 July 2020].

24 https://www.coe.int/en/web/data-protection/covid-19-data-protection-resources [22 July 2020].

25 Statement on the processing of personal data in the context of the COVID-19 outbreak. Adopted on 19 March 2020 https://edpb.europa.eu/sites/edpb/files/files/news/edpb_statement_2020_processingpersonaldataandcovid-19_en.pdf [25 July 2020].

26 Guidelines 04/2020 on the use of location data and contact tracing tools in the context of the COVID-19 outbreak Adopted on 21 April 2020, https://edpb.europa.eu/sites/edpb/files/files/file1/edpb_guidelines_20200420_contact_tracing_covid_with_annex_en.pdf [25 July 2020].

27 Guidelines 03/2020 on the processing of data concerning health for the purpose of scientific research in the context of the COVID-19 outbreak Adopted on 21 April 2020, https://edpb.europa.eu/sites/edpb/files/files/file1/edpb_guidelines_202003_healthdatascientificresearchcovid19_en.pdf [26 July 2020].

28 Statement on restrictions on data subject rights in connection to the state of emergency1 in Member States, adopted on 2 June 2020, https://edpb.europa.eu/sites/edpb/files/files/file1/edpb_statement_art_23gdpr_20200602_en.pdf [26 June 2020].

29 Statement on the data protection impact of the interoperability of contact tracing apps adopted on 16 June 2020, https://edpb.europa.eu/sites/edpb/files/files/file1/edpb_statementinteroperabilitycontacttracingapps_en_0.pdf [30 June 2020].

30 Statement on the processing of personal data in the context of reopening of borders following the COVID-19 outbreak adopted on 16 June 2020, https://edpb.europa.eu/sites/edpb/files/files/file1/edpb_statementreopeningbordersanddataprotection_en.pdf [30 July 2020].

31 *W. Wiewiórowski*, *EU Digital Solidarity, op. cit*. https://edps.europa.eu/sites/edp/files/publication/2020-04-06_eu_digital_solidarity_covid19_en.pdf [30 July 2020].

32 Orientations from the European Data Protection Supervisor, *op. cit*., https://edps.europa.eu/sites/edp/files/publication/20-07-15_edps_guidelines_remote_work_en.pdf [30 July 2020].

33 European Data Protection Supervisor Comments to DG Connect of the European Commission on the monitoring of COVID-19 spread. https://edps.europa.eu/sites/edp/files/publication/20-03-25_edps_comments_concerning_covid-19_monitoring_of_spread_en.pdf [30 July 2020].

34 Mobile applications to support contact tracing in the EU’s fight against COVID-19. Common EU Toolbox for Member States Version 1.0 of 15.04.2020, https://ec.europa.eu/health/sites/health/files/ehealth/docs/covid-19_apps_en.pdf [30 July 2020].

35 ehealth Network, Interoperability guidelines for approved contact tracing mobile applications in the EU, adopted on 13 May 2020 https://ec.europa.eu/health/sites/health/files/ehealth/docs/contacttracing_mobileapps_guidelines_en.pdf [20 July 2020].

36 eHealth Network Guidelines to the EU Member States and the European Commission on Interoperability specifications for cross-border transmission chains between approved apps, Detailed interoperability elements between COVID+ Keys driven solutions V1.0, adopted on 16 June 2020, https://ec.europa.eu/health/sites/health/files/ehealth/docs/mobileapps_interoperabilitydetailedelements_en.pdf [30 July 2020].

37 eHealth Network Guidelines to the EU Member States and the European Commission on Interoperability specifications for cross-border transmission chains between approved apps Basic interoperability elements between COVID+ Keys driven solutions V1.0, adopted on 12 June 2020, https://ec.europa.eu/health/sites/health/files/ehealth/docs/mobileapps_interoperabilityspecs_en.pdf [30 July 2020].

38 Commission implementing decision 2020/1023 of 15 July 2020 amending Implementing Decision (EU) 2019/1765 as regards the cross-border exchange of data between national contact tracing and warning mobile applications with regard to combatting the COVID-19 pandemic, https://eur-lex.europa.eu/legal-content/EN/TXT/PDF/?uri=CELEX:32020D1023&from=EN [15 September 2020].

39 https://ec.europa.eu/info/live-work-travel-eu/health/coronavirus-response/travel-during-coronavirus-pandemic/how-tracing-and-warning-apps-can-help-during-pandemic_en [9 November 2020].

40 Communication from the Commission, Guidelines on COVID-19 in vitro diagnostic tests and their performance (OJ 2020/C 122 I/01), https://eur-lex.europa.eu/legal-content/EN/TXT/PDF/?uri=CELEX:52020XC0415(04)&from=EN [30 July 2020].

41 EDPB Statement (...) adopted on 19 March 2020, *op. cit*. (Fn. 26).

42 *W. Wiewiórowski*, EU Digital Solidarity: *op. cit*., https://edps.europa.eu/sites/edp/files/publication/2020-04-06_eu_digital_solidarity_covid19_en.pdf [10 July 2020].

